# The Recent Yellow Fever Outbreak in San Antonio

**Published:** 1903-12

**Authors:** 


### THE RECENT YELLOW FEVER OUTBREAK IN SAN ANTONIO.

An impression seems to prevail in some parts of the State that the physicians of San Antonio were not united in their support of the Federal, State and local health authorities during the recent yellow fever episode in that city. The fact is that of ninety-eight or ninety-nine men engaged in the practice of medicine in San Antonio, more than eighty are outspoken in the belief that the disease in question was genuine yellow fever and are on record as having given State Health Officer Tabor, Dr. Murray and the local officials their hearty support and co-operation in every possible manner. Not more than five or six physicians denied positively the diagnoses made by Drs. Tabor and Murray, and the remaining ten or fifteen, not having investigated any of the cases were compelled to hold their opinions in reserve. It is true that the combined efforts of the five or six doctors in question resulted in complicating matters considerably, and in embarrassing the health authorities. Their efforts, however, were not seconded by any considerable part of the influential and conservative element of the citizenship, and the result is that the fever was easily brought under control to such an extent that Dr. Tabor was able to modify the quarantine at once, and, with the co-operation of the various neighboring county health officials, was soon able to raise it all together.

We do not mean to say that the gallant little band of "antis"

were left without support. One of the local papers, backed by a noisy and opinionated contingent, espoused their cause and joined energetically and right gladly in a campaign for the double purpose of vilifying the "yellow experts" and advertising the anti-fever doctors as "defenders of the people." The public was solemnly warned not to patronize any physician who admitted the existence of yellow fever. Grocerymen, clerks, boardinghouse keepers, Tom, Dick and Harry rushed into print to tell why they could not accept the diagnosis of yellow fever. The following head lines express alike the character of the writers, and of the paper giving them space to lay bare the poverty of their minds. "Who's the Liar," "A Bunch of Yellow Doctors Flock Around the House of Mr. W.," "Yellow Lie Nailed," "Rough on Experts," etc.

The anti-fever doctors were referred to as being possessed of all the manly virtues, and some of them responded by frequent letters to the people containing lengthy reviews of the situation, including detailed descriptions of the symptomalogy of the various cases under consideration, and learned dissertations upon yellow fever, malaria and dengue in general.

On November 13th the following "Honor Roll" was announced:

HONOR ROLL.

"The following physicians have defended San Antonio's good name against the calumnies of the yellow experts, and say most positively there has not been proven a single case of yellow fever in San Antonio this year. [I omit the names.—DANIEL.]

"The above physicians have plenty of gray matter under their hats, and no yellow streaks down their back bone.

"There are a number of other doctors who do not agree with the alarmists, but the iron hand of the medical trust is on their throats and they fear to speak."

Later the "Honor Roll" was again printed with the preface: "We take pleasure in recommending the following defenders of San Antonio." [Names omitted.—D.]

We will not believe that the names of these gentlemen were, with their knowledge and consent, placed before the public in such a connection. The yellow fever editor no doubt constructed the "Roll" on his own account for the purpose of securing, as he supposed, some advantage in the way of increased practice for the enrolled members at the expense of the "Yellow Fever Traitors," which simply goes to show what a low estimate the average layman places upon the principles of ethics adhered to by professional men in general, and by physicians in particular. W. B. RUSS.

